# Management of noma: practice competence and knowledge among healthcare workers in a rural district of Zambia

**DOI:** 10.1080/16549716.2017.1340253

**Published:** 2017-07-05

**Authors:** Mathilda Ahlgren, Tjede Funk, Clemence Marimo, Charlotte Ndiaye, Tobias Alfvén

**Affiliations:** ^a^ Global Health-Health Systems and Policy, Department of Public Health Sciences, Karolinska Institutet, Stockholm, Sweden; ^b^ School of Medicine, Cavendish University Zambia, Lusaka, Zambia; ^c^ Regional Advisor for Oral Health, WHO Regional Office for Africa, Harare, Zimbabwe; ^d^ Sachs’ Children and Youth Hospital, Stockholm South General Hospital, Stockholm, Sweden

**Keywords:** Noma, healthcare workers, knowledge, management, Zambia

## Abstract

**Background**: Noma is an infectious but opportunistic disease that often results in severe facial disfigurements and mortality if untreated. As noma progresses quickly, early detection and treatment are important to prevent its development.

**Objectives**: The objective of this study was to investigate primary healthcare workers’ knowledge and management of noma in a rural part of Zambia.

**Methods**: A cross-sectional self-completed survey was conducted among 35 healthcare workers from two district hospitals and 15 rural health centres in Serenje District, Zambia. Participants’ practice competences and knowledge were grouped into ‘optimal’, ‘medium’, ‘suboptimal’ and ‘very low’.

**Results**: Most of the healthcare workers stated that they perform mouth examination of a child below five years of age who is suffering from measles, malnutrition or HIV. A majority diagnosed gingivitis correctly and 40% had a medium level of practice competence of the same noma stage. All participants had a suboptimal or very low level on overall practice competence regarding management of noma and two-thirds had a very low level of reported knowledge.

**Conclusion**: General knowledge on noma and competences of diagnosing and treating noma patients was low among healthcare workers. Lack of knowledge could present a barrier for correctly managing noma at an early stage. Improving knowledge among healthcare workers is one way to prevent the development of the disease. In order to prevent noma from the start, actions need to be focussed on improving (oral) hygiene and health education as well. Telemedicine could also be considered as it can help healthcare workers in handling noma patients through enabling communication and exchange of information with specialist.

## Background

**Figure 1.**
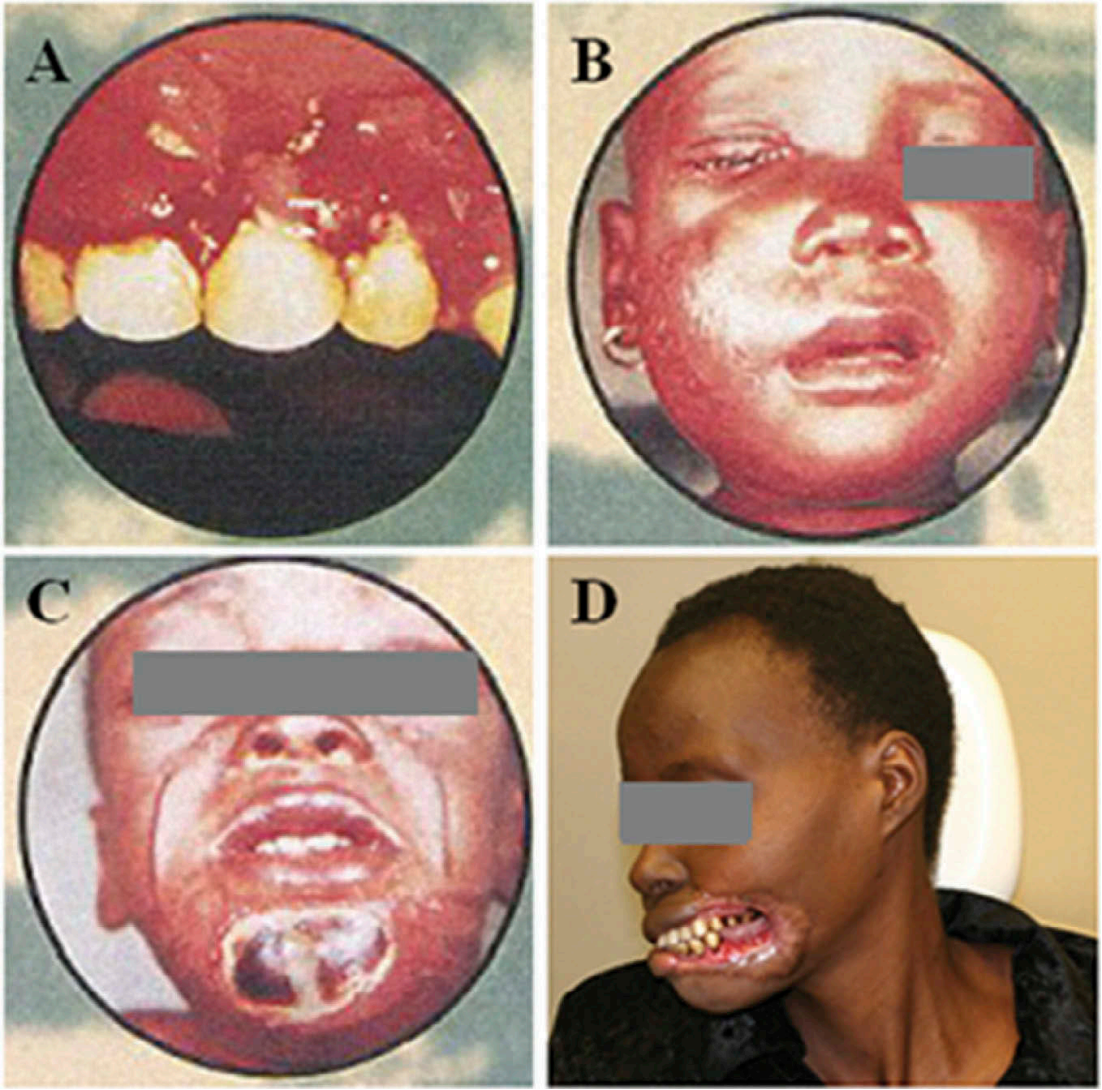
Examples of manifestations in the four different stages of noma. (a) Stage 1, gingiva is red, bleeding and has lesions. (b) Stage 2, cheek is swollen. (c) Stage 3, a gangrenous plaque has occurred at the chin. (d) Stage 4, gangrenous tissue of left lip, left commissure and left cheek has fallen off, leaving a hole. (a–c) Property of WHO/AFRO and (d) property of Clemence Marimo. Reproduced with permission.

Noma is an opportunistic infectious disease that mainly strikes children below the age of five. A combination of different factors leads to its development, poverty being most important [1–3]. Other important factors are malnutrition (both past and present), poor (oral) hygiene as well as measles and malaria [2,4,5]. The progress of noma is rapid and the disease can be said to consist of four different stages (Figure 1). The first stage involves bleeding gums with lesions, also called gingivitis. In the second stage, swelling of the cheek, chin or lips is seen, accompanied by fever. Within a few days a gangrenous plaque with a dark greyish and then black colour appears, which is considered to be the third stage. In the fourth stage, the gangrenous tissues have fallen off, leaving a hole in the affected part of the face [6,7]. Management and treatment of noma depends on its progress. While early stages can be treated with antibiotics, nutritional supplementation as well as improvements in oral hygiene, later stages require professional care and surgery [2,3,8]. Mortality rates, if untreated, are thought to be around 80–90%. However, access to treatment early on and provision of antibiotics reduces mortality rates to around 10–20% [5,9].

Noma occurs worldwide where extreme poverty prevails, but is most common in sub-Saharan Africa, in the so-called ‘noma belt’, a region stretching from Senegal to Ethiopia [1,10]. As accurate epidemiological data on noma are lacking worldwide, global incidence rate estimates vary between 40,000 and 140,000 cases per year [5,11,12].

There are no official figures on noma in Zambia (personal communication with Ministry of Health on 14 April 2009 and WHO Zambia on 16 April 2009), a country in which more than two-thirds of the population live in poverty and about 15% of children under five years are underweight and close to half are stunted [13,14]. To our knowledge, only one study regarding noma in Zambia exists, which describes 112 children with noma that were admitted to the University Teaching Hospital in Lusaka between 1979 and 1993 [15]. This is of course only the tip of the iceberg, as most noma cases never reach the hospital [1,6]. Thus, one can assume that prevalence rates are higher.

Although the risk of noma is small, measures can and should be taken to prevent this disease from developing. Early detection by health personnel prevents the disease from evolving as well as its complications. Consequently, mouth examinations and knowledge and awareness of early stages of noma can help to potentially lower the burden of the disease. The lack of knowledge among health personnel regarding noma has been discussed before [1,16], but to our knowledge no research exists that actually addresses the management and knowledge of noma among healthcare workers. The aim of this study, therefore, was to investigate primary healthcare workers’ knowledge and management of noma in a rural district of Zambia.

## Methods

### Study setting and participants

This study was carried out in Serenje District, Central Province, Zambia. Central Province was selected as it has a high prevalence of HIV as well as under-5 malnutrition [17]. Serenje District was chosen as it is a rural district, where noma is most likely to occur. At the time of data collection, the district had 17 health centres and two first-level hospitals (hospitals at district level which serve as the first referral level from health centres) [18]. The projected population for the year of 2009 was about 160,000 [19].

Healthcare workers were eligible to participate in this study if they (1) examined and/or treated patients seeking care at health centres or first-level hospitals in Serenje District and (2) were present at the health facilities at the time of data collection.

### Data collection

Data were collected between March and April 2009. A self-completed questionnaire was developed together with experts on noma based on the WHO manual for noma. A pre-test of the questionnaire took place in Zambia outside the study area.

The two hospitals and all health centres within the Serenje district were visited except for one health centre which was not reachable during the study period due to flooding. At one health centre there was no healthcare worker present at the time of data collection. Thus, healthcare workers of two hospitals and 15 health centres were included in this study. Each facility was visited once, except Serenje hospital which was visited twice to cover personnel working on different shifts.

Every study participant filled out the questionnaire which contained both closed and open-ended questions. The questionnaire consisted of two parts. The first part contained participants’ demographic information as well as questions on the number of (child) patients they treat, the number of patients with oral health problems, participants’ mouth examination practice and simulated cases on noma. The part on simulated cases represented one scenario for each of the four stages of noma (Figure 1). In each scenario, participants needed to answer four questions: what they would ask the patient/care-giver, what the probable diagnosis is, what treatment they would give and what advice they would give to the patient. The second part of the questionnaire addressed knowledge on noma directly. This part included questions on knowing noma, its risk factors, consequences of untreated or poorly managed noma as well as its treatment and prevention (see Supplementary Material 1).

The questionnaire was filled in without any aid but under supervision. Up to five persons did this in the same sitting. The time for completing the questionnaire varied from 20 to 40 minutes. The participants were informed that it was a study about oral health. The first part of the questionnaire, including general information and case scenarios, was distributed first. No information that it was a study regarding noma was revealed before the second part of the questionnaire was handed out. This part was only distributed when the previous part of the questionnaire was handed in.

### Data analysis

The answers from the case scenarios and the open-ended questions were coded and categorized into either correct or incorrect according to WHO’s manual on noma and the literature. The practice competence of participants was calculated by scoring the answers given to the four noma case scenarios. The highest total practice competence score was 18. For each noma case scenario a total score of 4.5 could be achieved if the four questions asked about the scenario were answered correctly; 1.5 points for questions asked to parent and 1 point for the correct diagnosis, treatment and advice given, respectively. Multiple answers were required for all questions (except diagnosis). Each correct answer equalled 0.25 points, only correctly mentioning referral under the question on treatment gave 0.5 points due to its very high importance. Consequentaly, depending on the number of answers required for each question, different weights for the questions emerged. For a detailed description of the scoring system see Supplementary Material 1B.

For the part on knowledge on noma, a total score of 5.8 could be achieved. Risk factors corresponded to 1 point (correctly mentioning HIV, malnutrition, poor hygiene and infection were each worth 0.25 points). There were three correct answer options for consequences of untreated and consequences of poorly managed nome, namely death, sequalae and stigma. In both questions each of these answers was equivalent to 0.3 points. If all four managing practices were correctly identified (mouth rinses, local disinfection, antibiotics and additional feeding with vitamins), 1 point was given. Finally, correct knowledge on how noma can be prevented at work as well as in society corresponded to 1 point each.

The final practice competence score as well as knowledge score was then divided into three groups, optimal (≥ 75% of total score), medium (50–74% of total score) and suboptimal (< 50% of total score). These limits have been used as cut-off points in previous studies about knowledge among health personnel [20,21]. A fourth group, very low (< 25%), was later added to distinguish the knowledge level within the suboptimal group. Data were analysed using SPSS software (version 16.0) for descriptive statistics and chi-square test to determine possible correlations. *P*-value < 0.05 was used as level of significance.

### Ethical issues

The study was approved by the Biomedical Research Ethics Committee (No. FWA00000338) of the University of Zambia School of Medicine on 18 March 2009. Approval to conduct the study was also given by the Ministry of Health, Zambia and by the Director of Health in Serenje District. All respondents were informed that participation was voluntary, anonymous and that it was not a test. Written consent was obtained from all participants before distribution of questionnaires.

## Results

### General information

Of all healthcare workers asked to participate, two declined participation. The response rate was thus 95% (*n *= 35/37; district hospital *n* = 13, rural health centre *n* = 22). The respondents’ mean age was 34.5 years with a range from 23 to 55 and 66% were 35 years old or younger (Table 1). All had an education at college level with one at university level. There were more males (57%) than females and 63% of the respondents worked at a health centre. Participants were clinical officers, nurses, dental therapists and environmental health technicians. Health workers’ estimation on how many patients under 5 years they see per day ranged between 0 and 45 (mean: 18, median: 15).

**Table 1.** Sociodemographic characteristics of study participants.

Characteristics	District hospital(*n *= 13)	Rural health centre(*n *= 22)	Total(*n *= 35)
**Sex**			
Men	4	16	20
Women	9	6	15
**Age group (years)**			
≤ 35	11	12	23
> 35	2	10	12
**Provider types**			
Clinical officers	0	6	6
Nurses	12	13	25
Dental therapists	1	0	1
Environmental health technicians	0	3	3
**Mean of self-estimated number of patients under 5 years of age that each healthcare worker cares for per day**	11	23	18

Out of all respondents, four reported that they had treated patients with noma, the number of patients ranging from one to six.

### Conduction of mouth examinations

**Figure 2.**
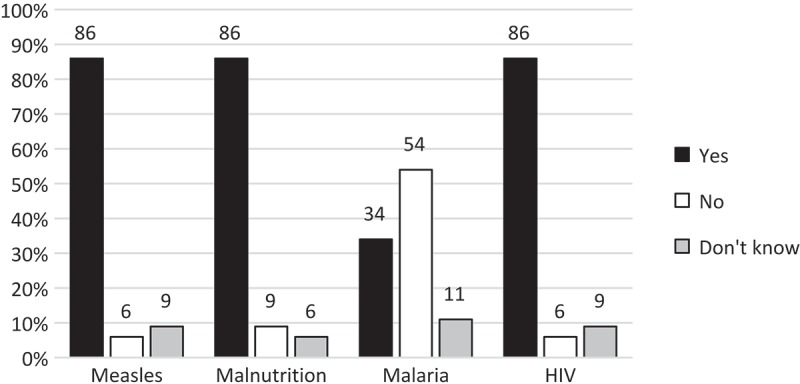
Frequency of mouth examination (*n* = 35). Responses from 35 healthcare workers to the question about performance of mouth examination of a child under five suffering from different conditions that are known to be risk factors for noma (percentages rounded, therefore not always total 100%).

Most of the healthcare workers (86%) stated that they perform mouth examinations in a child under five years who is suffering from measles, malnutrition or HIV (Figure 2). For malaria this percentage was lower, with only one-third of the participants reporting that they examine the mouth of the child in this case.

### Practice competence regarding management of noma

The responses to diagnoses and treatment in the different stages are shown in Table 2. Most participants diagnosed the first stage of noma correctly, whereas one person had written noma and 69% gingivitis. The second stage of noma was the stage that was least correctly diagnosed, by only 6% of the healthcare workers. Regarding treatment, the proportion that correctly identified antibiotics was 49–66% for the different noma stage scenarios. Local disinfection was the treatment missed out mostly. A minority mentioned referral in the third and fourth stage (14% and 31%, respectively).

**Table 2.** Number and percentage of respondents that correctly identified the right diagnose and treatment for the different stages of noma.

Correctly identifying	Total (*n* = 35)
**Diagnosing**	
Noma stage 1/gingivitis	25 (71%)
Noma stage 2	2 (6%)
Noma stage 3	7 (20%)
Noma stage 4	10 (29%)
**Treatment**	
*Stage 1*	
Local disinfection	2 (6%)
Mouth rinses	15 (43%)
Antibiotics	20 (57%)
Additional feeding with vitamins	4 (11%)
*Stage 2*	
Local disinfection	0 (0%)
Mouth rinses	8 (23%)
Antibiotics	17 (49%)
Additional feeding with vitamins	7 (20%)
*Stage 3*	
Local disinfection	2 (6%)
Antibiotics	19 (54%)
Referral	5 (14%)
*Stage 4*	
Local disinfection	1 (3%)
Antibiotics	23 (66%)
Referral	11 (31%)

**Figure 3.**
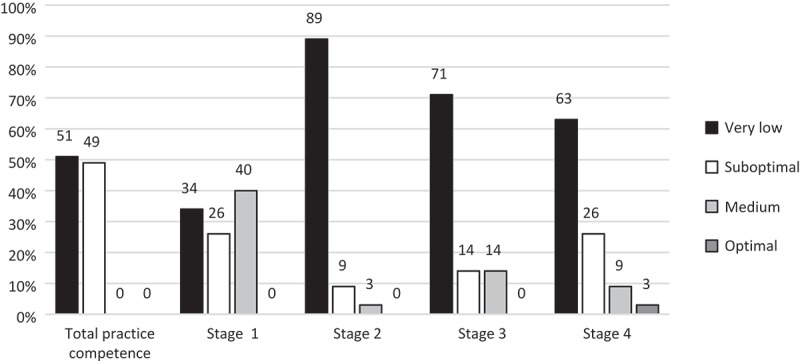
Practice competence regarding management of noma (*n* = 35).

Figure 3 shows that all participants had a suboptimal or very low overall practice competence. The best practice competence was found in stage one with 40% scoring medium, compared to 3% in stage 2, 14% in stage 3 and 3% in stage 4. The second stage of noma, with nearly 100%, had the highest proportion of suboptimal and very low practice competence scores.

### Reported knowledge on noma

In total, 54% of the respondents stated that they know about noma. Among these, about half had got to know about noma during education and half through work. All respondents but one stated that they had not participated in any noma training programme. The respondent who did had seen a video film about noma during education.

**Figure 4.**
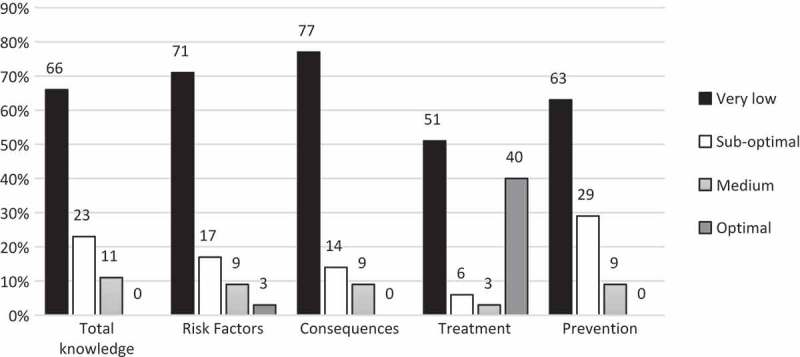
Reported knowledge on noma (*n* = 35).

Reported knowledge on noma was poor (Figure 4). No one had optimal knowledge and only 11% had medium knowledge levels. The proportion of participants who had very low or suboptimal knowledge was 88% for risk factors, 91% for consequences of untreated or poorly managed noma, and 92% for prevention. Treatment was the subject where respondents scored highest, with 40% having optimal knowledge. There were no statistical significant differences between healthcare workers at health centres and hospitals either in reported knowledge or in subgroups.

## Discussion

The results of this study show that most of the healthcare workers (86%) perform mouth examinations on children with malnutrition, measles and HIV, which are the most important predisposing factors for noma. This study shows that not only do a majority of healthcare workers conduct mouth examinations, but also that 71% recognize gingivitis, an early stage of noma. The management of this stage of noma is also reasonable. In addition, most respondents (49–66% for the different scenarios) correctly identified antibiotics as treatment when needed.

However, knowledge about noma was limited. All participants had a very low or suboptimal level on total practice competence and two-thirds had a very low level of reported knowledge. Knowledge was particularly lacking for the second stage with the highest frequency of very low practice competence (89%). The development of noma is very rapid, but at this stage it is still possible to prevent noma and its sequelae from developing further by giving effective antibiotics together with local disinfection and nutritional rehabilitation [6].

Knowledge and treatment/referral practices of healthcare workers for the third and fourth stages of noma were very limited. Only 14% in stage 3 and 31% in stage 4 of healthcare workers reported to refer the patient. During these stages referral and correct treatment are absolutely necessary, as untreated cases of noma are most often fatal [6,9].

Hence, the data from this study show that a lack of knowledge among healthcare workers regarding noma exists. However, even with this very low knowledge regarding noma, its causes, background and consequences, most of the healthcare workers look into the mouth of the patients, recognize gingivitis and many use antibiotics when needed.

As Challacombe et al. [22] present, actions against noma can be taken on different levels. At the primary healthcare level, education of health personnel is one way to improve early recognition and referral of noma cases. Despite noma being a very devastating disease, it is still uncommon. Considering also the existence of a number of other neglected diseases, educating all front-line healthcare workers on all aspects of noma might not be a realistic approach. More feasible could be to focus on the promotion of oral examinations and training to improve knowledge and practices of early stages of noma, such as gingivitis. These practices lead to an earlier detection and more rapid provision of care, both being important in the prevention of noma [23]. Furthermore, these prevention practices can be handled at health centre level without need of referral to specialized hospitals. The most important thing might thereby not be to be able to diagnose noma as such, but to recognize when something needs to be done and to know how to treat it correctly or when to refer cases. It is important that not only dentists and dental therapists are addressed, but all other healthcare workers who examine and treat patients.

Besides introducing and strengthening national and community programmes against noma, telemedicine could be considered for the management of noma. As the WHO recognizes, telemedicine has a great potential. In low-income countries it is mostly used for connecting healthcare workers with specialists [24]. Evidence shows that mobile phones can facilitate diagnosis, for instance through taking pictures [25,26]. The power of mobile images can be helpful in the diagnosis of noma. Healthcare workers can then use phones for communication and information exchange with staff from other health facilities, such as specialized hospitals, in cases where they are unsure about diagnosis and treatment [26]. Although the introduction of telemedicine interventions comes with many obstacles and challenges, advantages are plenty, such as knowledge exchange, information dissemination and providing support to healthcare workers [24]. This study showed that referral practices were low, although life-saving in the late stages of noma. Introduction of telemedicine or other methods of information exchange techniques between health professionals may help in addressing this problem.

There was no statistically significant difference between healthcare workers at health centres and hospitals either regarding practice competence or knowledge on noma. This shows that knowledge is lacking equally in both health facilities, showing that future interventions should address different levels. Other correlations were not tested due to the relatively small size of the study.

One contributing factor to the low knowledge level among healthcare workers in this study could be that noma cases are rare. Further, it has been shown before that only around 10% of care-givers seek medical care in the acute phase [6,16]. Noma is also strongly connected with traditional beliefs and stigma [15,27,28]. It is therefore possible that noma patients do not seek care and, thus, healthcare workers never get to see an affected person, even though they exist in the community. The fact that four healthcare workers in this study said they have treated patients with noma and nine reported that they have learnt about noma through work suggests that noma does occur within this area. However, there might be other places in Zambia that have a higher prevalence of noma due to being more remote and having different nutritional habits [15].

### Strengths and weaknesses

The strengths of this study are that every health facility in the study district at the time of the study was visited except one. Additionally, the response rate was high, which is why this study covers the district well. The study also provides new findings as, according to our knowledge, there is no previous study done on knowledge and practice competence among healthcare workers regarding noma.

Zambia has a high prevalence of HIV and under-five malnutrition [14], and therefore an increased risk for noma. The majority of health facilities within the country are rural health centres and Serenje District might therefore represent a larger proportion of the country when it comes to risk for noma and healthcare workers’ management and knowledge about it.

Although the whole district was covered, the size of the study was relatively small. Another limitation is that the practice competence and knowledge scores are based on self-reporting and might therefore not reflect the actual practice of the respondents regarding noma. This means that actual management and treatment practices could be even lower. Accessibility to clear guidelines regarding recommended treatment and types and dosage of antibiotics would ease the management of noma.

## Conclusion

Practice competences and general knowledge on noma was low among healthcare workers at both district hospitals and rural health centres. Nevertheless, a majority did conduct oral examinations in children with malnutrition, measles and HIV and also recognized gingivitis (the first stage of noma). Health personnel’s lack of knowledge on noma could be one constraint in the prevention of noma. Although this study recognized this fact, only educating healthcare workers on noma, a still uncommon disease, might not be the best and most efficient way to prevent noma. Actions against noma should not only focus on the education of healthcare workers, but also on community actions, such as health education and promotion of (oral) hygiene. The introduction of telemedicine could also be considered. The fact is, however, that noma is a devastating disease with tremendous consequences which can be prevented. Therefore, actions need to be taken on different levels to prevent and eradicate it.

## Supplementary Material

Supplemental Data

## Paper context

Noma is a rare and devastating disease, mainly affecting young children. If not recognized and treated in a timely manner, noma is fatal in most cases. Health workers’ knowledge about noma and their practice competence in handling noma cases is crucial for management of the disease. This study from Zambia examines to what extent health workers recognize and treat the condition correctly and have knowledge of noma.
