# Supplementary material for: Management of noma: practice competence and knowledge among healthcare workers in a rural district of Zambia
Source: Glob Health Action. 2017 Jul 5;10(1):1340253. doi: 10.1080/16549716.2017.1340253 (PMC5533138; doi:10.1080/16549716.2017.1340253)
Supplement: Supplemental Data [file zgha_a_1340253_sm1909.docx]

SUPPLEMENTARY MATERIAL 1

| ID Respondent Type of health worker Date (dd/mm/yyyy)............/........../2009 | Health worker code 1=Medical doctor 3= Dental therapist 2=Nurse 4= Other |
| --- | --- |

**General information**

1. Age [in years] .......................
2. Sex (Please circle): 1=Male 2=Female
3. Education level (Please circle one correct answer)
   1=Grade 7 4=College
   2=Grade 9 5=University
   3=Grade 12 6=Postgraduate
4. Occupation (Please circle one correct answer)
   ***A. For Medical doctors***1=Medical specialist (specify field and year of graduation..........................................)
   2=Post-graduate (specify field and year of graduation..........................................)
   3=Medical doctor (year of graduation..........................)
   4=Clinical officer (year of graduation..........................)
   ***B. For Nurses***1=Nurse (Please specify the duration of learning ............years)
   2=Post basic Qualification (Please specify the duration of learning ............years)
   3=Auxiliary nurse (Please specify the duration of learning ............years)
   ***C. For other Health workers***Please specify occupation and the duration of learning ......................./.............years
5. Workplace (Please circle correct answer, more than one option possible)
   1=Provincial/central hospital
   2=District hospital
   3=Private clinic
   4=Health centre
   5=Health post
   6=Other (Please specify....................................................)
6. How many patients do you take care of per day, in average? ..........................................
7. About how many of these are children below five years of age? ....................................
8. About how many of the patients that you take care of every month are children seeking care due to oral health problems?

...........................................................................................................................................

1. How many of these children have problems with their teeth? ...........................................................................................................................................
2. How many of these children have problems with their gums? ...........................................................................................................................................
3. Is it possible that parents seek care for their children due to these problems somewhere else than at your workplace? (Please circle one answer)

1=Yes 2=No 3=Don’t know
If yes, please specify where .............................................................................................
..........................................................................................................................................

1. If a parent seeks care for a child below 5 years of age due to the problems stated below, would you then examine the mouth? (Please circle one answer for each option)

1=Measles 1=Yes 2=No 3=Don’t know
2=Malnutrition 1=Yes 2=No 3=Don’t know
3=Malaria 1=Yes 2=No 3=Don’t know
4=HIV 1=Yes 2=No 3=Don’t know

*Turn to next page please.*

**Simulated cases**

1. A mother seeks care for her 2-year old child who has bleeding gums and foul breath.

   A. What questions would you ask the mother?
   ......................................................................................................................................................................................................................................................................................
   ...........................................................................................................................................

   B. What would be the probable diagnosis?
   ......................................................................................................................................................................................................................................................................................
   ...........................................................................................................................................

   C. What treatment would you give the child for that probable diagnosis?
   ......................................................................................................................................................................................................................................................................................
   ...........................................................................................................................................

   D. What advice would you give?
   ......................................................................................................................................................................................................................................................................................
   ...........................................................................................................................................
2. You have a 4 year old patient with fever and swollen lips and cheek.

   A. What questions would you ask the care-giver?
   ......................................................................................................................................................................................................................................................................................
   ...........................................................................................................................................

   B. What would be the probable diagnosis?
   ......................................................................................................................................................................................................................................................................................
   ...........................................................................................................................................

   C. What treatment would you give the child for that probable diagnosis?
   ......................................................................................................................................................................................................................................................................................
   ...........................................................................................................................................

   D. What advice would you give?
   ......................................................................................................................................................................................................................................................................................
   ...........................................................................................................................................
3. A parent comes with a 3-year old child whose chin has a dark, greyish/black mark with partly gangrenous plaque.

   A. What questions would you ask the care-giver?
   .................................................................................................................................................................................................................................................................................................................................................................................................................................

   B. What would be the probable diagnosis?
   ......................................................................................................................................................................................................................................................................................
   ...........................................................................................................................................

   C. What treatment would you give the child for that probable diagnosis?
   ......................................................................................................................................................................................................................................................................................
   ...........................................................................................................................................

   D. What advice would you give?
   .................................................................................................................................................................................................................................................................................................................................................................................................................................
4.
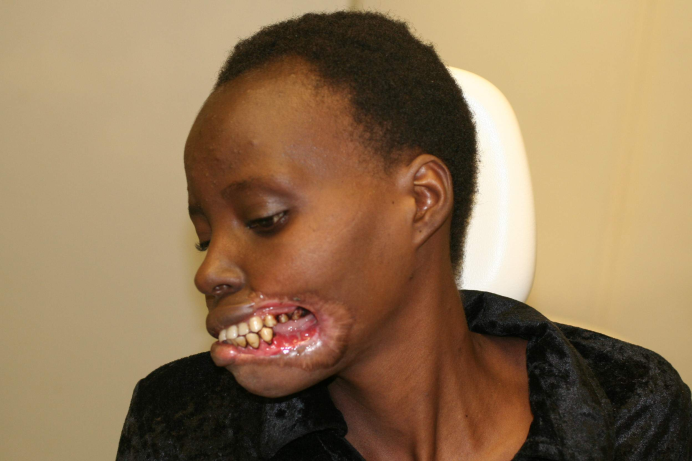
You get a 25-year old patient with gangrenous tissue and a hole in her cheek.


   A. What questions would you ask the patient?
   .................................................................................................................................................................................................................................................................................................................................................................................................................................

   B. What would be the probable diagnosis?
   ......................................................................................................................................................................................................................................................................................
   ...........................................................................................................................................

   C. What treatment would you give the patient for that probable diagnosis?
   ......................................................................................................................................................................................................................................................................................
   ...........................................................................................................................................

   D. What advice would you give? ......................................................................................................................................................................................................................................................................................
   ...........................................................................................................................................

**Knowledge and practice on noma** ID Respondent: .

1. Do you know about noma (cancrum oris)? (Please, circle one answer)
   1=Yes 2=No

If yes,
A. When did you first get to know about noma? .............................................................

B. How did you first get to know about noma? (Please, circle one correct answer)
a) During education
b) Through media
c) Through your work
d) Other, please specify....................................................................................................

1. Please list the risk factors for noma that you know
   .................................................................................................................................................................................................................................................................................................................................................................................................................................
2. What are the consequences of untreated noma?
   .................................................................................................................................................................................................................................................................................................................................................................................................................................
3. What are the consequences of poorly managed noma?
   .................................................................................................................................................................................................................................................................................................................................................................................................................................
4. What should always be done in managing noma? (Please circle one answer for each option)

1=Mouthrinses 1=Yes 2=No 3=Don’t know
2=Give as much cassava as possible 1=Yes 2=No 3=Don’t know

3=Local disinfection 1=Yes 2=No 3=Don’t know
4=Provide antibiotic therapy 1=Yes 2=No 3=Don’t know
5=Give additional feeding with vitamins 1=Yes 2=No 3=Don’t know

1. Can noma be prevented? (Please circle one answer)
   1=Yes 2=No 3=Don’t know
   If yes,
   A. How to prevent noma in your work?
   ............................................................................................................................................................................................................................................................................................................................................................................................................................................................................................................................................................................ B. How to prevent noma in society?
   ............................................................................................................................................................................................................................................................................................................................................................................................................................................................................................................................................................................
2. Have you ever treated a patient with noma? (Please circle one answer)
   1=Yes 2=No 3=Don’t know
   If yes, how many (approximately)? .............................
3. Have you attended any training or educational program of noma? (Please circle one answer)
   1=Yes 2=No
   If yes, please specify ......................................................................................................................................................................................................................................................................................

*Thank you very much for your attention and participation!*

SUPPLEMENTARY MATERIAL 1B

**Scoring system**

**Practice Competence Score**

The practice competence scores were calculated based on four simulated noma cases.

For each noma case scenario participants needed to know the following:

1. **What questions would you ask the mother?**

Correct answers: history taking (0.25 points), symptoms (0.25 points), asking about malnutrition (a risk factor for noma; 0.25 points), asking about oral hygiene (a risk factor for noma; 0.25), asking about HIV (a risk factor for noma; 0.25), previous treatment (0.25 points)

Maximum score for question 1: 1.5 points

1. **What would be the probable diagnosis?**

Correct answers: Stage 1: noma OR gingivitis (1 point), Stage 2, Stage 3 and Stage 4: noma (1 point).

Maximum score for question 2: 1 point

1. **What treatment would you give the child for that probable diagnosis?**

Correct answers: For stages 1 and 2: local disinfection (0.25 points), mouth rinses (0.25 points), antibiotics (0.25 points), additional feeding with vitamins (0.25 points)

For stages 2 and 4: local disinfection (0.25 points), antibiotics (0.25 points), referral (0.5 points)

NOTE: referral was weighted with 0.5 points because it is the most crucial in the treatment of noma Stages 3 and 4

Maximum score for question 3: 1 point

1. **What advice would you give?**

Correct answers: Stages 1 and 2: nutritional advice (0.25 points), advice on general health (0.25 points), advice on oral health (0.25 points), immunisation (0.25 points)

Stages 3 and 4: Bring siblings for assessment (0.25 points, nutritional advice (0.25 points), advice on general health (0.25 points), advice on general health (0.25 points)

Maximum score for question 4: 1 point

**TOTAL achievable practice competence score: 18 points (4,5 points for each scenario, ie. 4,5 x4)**

Optimal practice competence= ≥75% of total score
Medium practice competence = 50 – 74% of total score
Suboptimal practice competence = 25-49% of total score
Very low practice competence = >25% of total score

**Knowledge score**

The knowledge score for this study was calculated based on the part 2 of the questionnaire with specific questions on noma.

1. **Please list the risk factors for noma that you know**

Correct answers: malnutrition (0.5 points), poor hygiene (0.25 points), infection (0.25 points), HIV (0.25)

Maximum score for question 1: 1 point

1. **What are consequences of untreated noma?**

Correct answers: death (0.3 points), sequalae (0.3), stigma (0.3)

Maximum score for question 2: 0.9 points

1. **What are the consequences of poorly managed noma?**

Correct answers: death (0.3 points), sequalae (0.3), stigma (0.3)

Maximum score for question 3: 0.9 points

1. **What should always be done in managing noma? (Please circle one answer for each option)**

1= Mouth rinses 1=yes 2=No 3=Don’t know

2= Give as much cassava as possible 1=yes 2=No 3=Don’t know

3= Local disinfection 1=yes 2=No 3=Don’t know

4= Provide antibiotic therapy 1=yes 2=No 3=Don’t know

5= Give additional feeding with vitamins 1=yes 2=No 3=Don’t know

Circling YES for mouth rinses, local disinfection, antibiotics and additional feeding with vitamins gave each 0.25 points. Incorrectly circling an option gave 0 points.

Maximum score for question 4: 1 point

1. **How to prevent noma in your work?**

Correct answers: gingival status (0.25 points), nutrition information (0.25 points), hygiene information (0.25 points), advice VCT (0.25 points)

Maximum score for question 5: 1 point

1. **How to prevent noma in society?**

Correct answers: nutrition and hygiene improvements (0.25 points), fight stigma (0.25 points), inform caretakers (0.25 points), immunisation programmes (0.25 points)

Maximum score for question 6: 1 point

**TOTAL Knowledge score: 5.8 points**

Optimal practice competence= ≥75% of total score
Medium practice competence = 50 – 74% of total score
Suboptimal practice competence = 25-49% of total score
Very low practice competence = >25% of total score
